# Lifespan and functionality of mycorrhizal fungal mycelium are uncoupled from host plant lifespan

**DOI:** 10.1038/s41598-018-28354-5

**Published:** 2018-07-06

**Authors:** Alessandra Pepe, Manuela Giovannetti, Cristiana Sbrana

**Affiliations:** 1 Department of Agriculture, Food and Environment, University of Pisa, Pisa, 56124 Italy; 2 CNR-Institute of Agricultural Biology and Biotechnology, UOS Pisa, Pisa, 56124 Italy

## Abstract

Arbuscular mycorrhizal fungi (AMF) are obligate symbionts, living in associations with the roots of most land plants. AMF produce wide networks of extraradical mycelium (ERM) of indeterminate length, spreading from host roots into the surrounding soil and establishing belowground interconnections among plants belonging to the same or to different taxa. Whether their lifespan and functionality are limited by host plant viability or can be extended beyond this limit is unknown. To address this issue, we performed time-course studies to investigate viability and functionality of ERM produced in an *in vivo* whole-plant system by *Funneliformis mosseae* and *Rhizoglomus irregulare*, after shoot detachment. Our data revealed that viability and functionality of *F. mosseae* and *R. irregulare* extraradical hyphae were uncoupled from host plant lifespan. Indeed, ERM spreading from roots of intact or shootless plants showed comparable levels of viability, similar structural traits and ability to establish mycorrhizal symbioses with new plants, as long as five months after shoot removal. Our findings expand the current knowledge on AMF biology and life cycle, providing data on ERM long-term survival in the soil of two Glomeracean species, functional to the prompt establishment of mycorrhizal symbioses and to the maintenance of soil biological fertility.

## Introduction

Arbuscular mycorrhizal fungi (AMF, Glomeromycotina) are beneficial soil symbionts establishing mutualistic associations with the roots of 80% of plant species and the large majority of food crops, including cereals, legumes, vegetables and fruit trees. AMF are key elements of soil fertility, which depends on biological, chemical and physical components, of plant nutrition and productivity, absorbing soil mineral nutrients by a fine network of extraradical hyphae growing from colonised roots into the soil^1^ and delivering them to host plant roots, where a reciprocal nutrient exchange occurs through arbuscules, intracellular fungal branched structures. Many works support the obligate biotrophy of AMF for carbohydrates and their inability to synthesize fatty acids, suggesting that such carbon sources may play an important role in regulating AMF intraradical proliferation, arbuscule development and life cycle completion^2^. Belowground mycorrhizal networks extend the absorbing surface area (up to 40 times) growing in every direction^3,4^, efficiently exploring the soil and increasing plant uptake of phosphorus, nitrogen, sulphur, immobile micronutrients such as copper and zinc and other soil-derived mineral cations^5–9^. In addition, AMF protect plants from biotic and abiotic stresses, such as pathogens, drought and salinity^10–13^ and affect plant secondary metabolism, enhancing the synthesis of beneficial phytochemicals, thus contributing to the sustainable production of high-quality food^14,15^.

Several molecular studies investigated the functioning of extraradical mycelial networks (ERM) and revealed that genes encoding proteins for transport of mineral nutrients, such as phosphorus, zinc, nitrogen, are differentially expressed in ERM hyphae, thus confirming their key role in mineral uptake in the soil-fungus interface^16,17^. Other studies investigated the extent and interconnectedness of AMF hyphal networks, which represent critical factors for the maintenance of nutrient flow from the extraradical to the intraradical phase. ERM density is estimated to range from 2.7 to 20.5 m/g of soil^18,19^, with a mean growth rate of 0.74–1.1 m d^−1^ and a specific weight of 3.8–7.8 μg m^−1 ^^3,20^. ERM formed by members of the family Glomeraceae, the most abundant AMF in agricultural soils, are highly interconnected by means of fusions (anastomoses) between contacting hyphae, reaching the value of 100–410 anastomoses per gram of soil^18,19,21^. Such structural traits are of functional significance for the uptake, translocation and flow of nutrients from soil to host roots, as ERM extent and interconnectedness have been shown to be positively correlated with host growth response variables and P content, affecting symbiotic performance and plant growth and nutrition^22^.

So far, ERM ability to survive and maintain infectivity independently from the host plant lifespan have not been adequately investigated, although the fate and behaviour of dead colonized roots and their connected extraradical hyphae after host plant harvest may affect the production of spores, fundamental for the survival of AMF communities in the soil. The conservation of the whole soil mycorrhizal potential, represented by colonized roots, ERM and spores, represent an essential factor of soil biological fertility in organic and sustainable food production systems^23–25^. The role of plant root cell death in determining the viability of AM fungal symbionts has been long debated, and previous studies showed that, beyond spores, both living and dead root fragments can represent a source of inoculum for newly developing roots^23,26–28^. Notwithstanding, the question on the viability and lifespan of extraradical fungal hyphae after the detachment of root system from the host plant shoot has remained long unanswered.

The aim of the present work was to investigate ERM viability and functionality in shootless mycorrhizal plants, after assessing viability loss of roots. To this aim, an *in vivo* whole-plant experimental system and two worldwide distributed Glomeracean AMF, *Funneliformis mosseae* and *Rhizoglomus irregulare*, were utilised. On a time-course basis, after removing the shoots of the host plants, we monitored for 5 months (i) ERM structural traits and viability by assessing cellular succinate dehydrogenase activity, (ii) ERM maintenance of functionality by testing its ability to establish mycorrhizal symbioses in young seedlings placed in contact with ERM of different ages.

## Results

### Viability of plant roots after shoot removal

**Figure 1:**
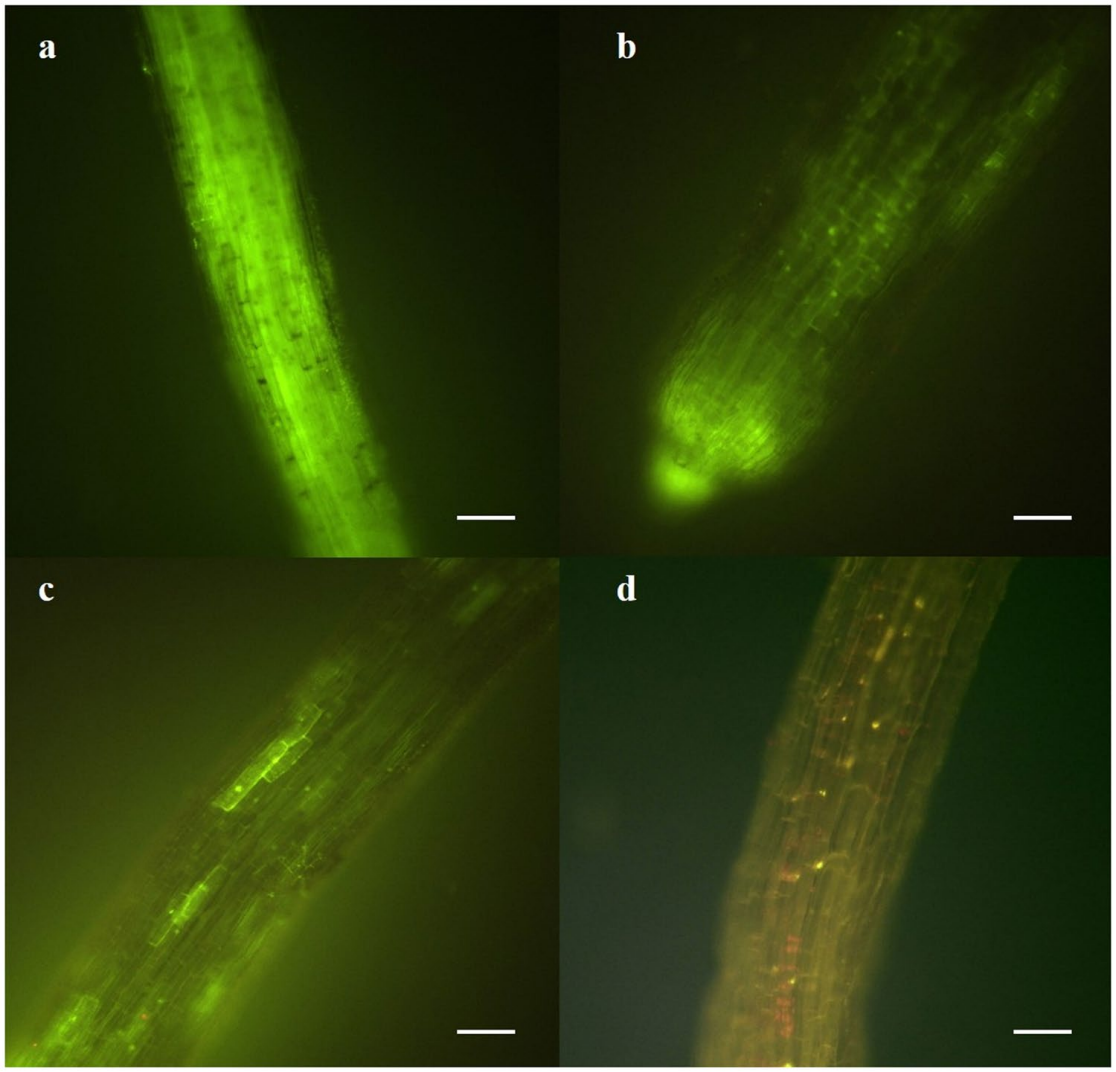
Fluorescein diacetate staining showing viability of *Cichorium intybus* roots, 1 (**a**), 2 (**b**), 3 (**c**) and 4 (**d**) days after shoot detachment from roots; scale bars = 120 µm (**a**) and 90 µm (**b**–**d**).

After FDA staining carried out in Experiment 1, roots of shootless *C. intybus* showed viable length rates of 55 ± 3% and 30 ± 2%, 24 h and 48 h after shoot removal, respectively. On the third day, 14 ± 1% viable length was detected, mainly at the root tips, whereas on the fourth day all the roots were not viable (Fig. 1). No significant differences were found in the decrease of viability between mycorrhizal and non mycorrhizal root systems after their detachment from shoots (24 h: F_1,4_ = 0.95, P = 0.39; 48 h: F_1,4_ = 5.54, P = 0.08; 72 h: F_1,4_ = 0.22, P = 0.66; 96 h: viable root length was 0 in both mycorrhizal and non mycorrhizal root systems).

**Figure 2:**
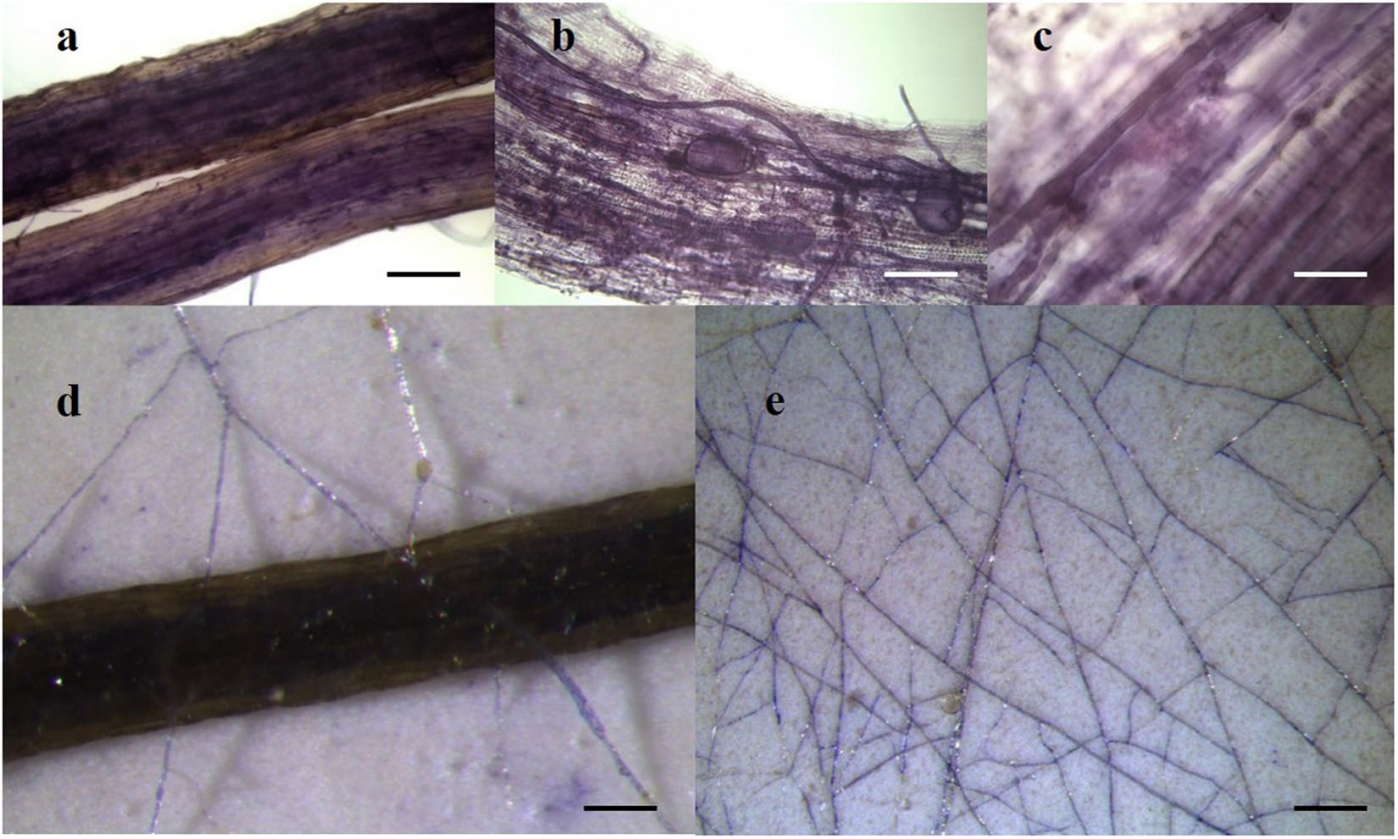
Membranes bearing *Cichorium intybus* roots colonised by *Funneliformis mosseae* IMA1 and *Rhizoglomus irregulare* IMA6, stained for succinate dehydrogenase (SDH) activity 4 months after shoot detachment. (**a**,**b**) Dead roots with SDH-positive intraradical fungal structures; scale bars = 120 µm (**a**) and 70 µm (**b**). (**c**) Viable (SDH-positive) arbuscule within a dead root cell; scale bar = 35 µm. (**d**) Viability of IMA1 extraradical hyphae emerging from a dead colonised root indicated by the deposition of blue formazan salts; scale bar = 100 µm. (**e**) SDH-positive extraradical network of IMA1 spreading on the membrane; scale bar = 120 µm.

At each time point of Experiment 2 (Exp. 2), SDH localisation did not reveal viable cells in root systems of shootless plants (Fig. 2a,d), although SDH-positive arbuscules were consistently detected, up to the end of the experiment (Fig. 2b,c).

### Length of ERM spreading from roots after shoot removal

Results of Exp. 2 showed that the membrane area covered by ERM at the time of shoot removal ranged between 12 and 15 cm^2^, and mycelial lengths were similar among the two AMF tested (Table 1, time 0). Total length of ERM growing from roots of shootless or intact plants (treatments) was comparable at any time point after shoot removal in both AMF isolates (Table 1). Three-ways ANOVA with fungal identity*treatment (shoot detachment/not)*time showed that time and fungal identity were significant factors affecting ERM total and viable length, with significant interaction between time and shoot removal treatment and between time and fungal identity (time F_23,48_ = 78.3, P < 0.001; AMF F_23,48_ = 7.6, P = 0.008; time x AMF F_23,48_ = 9.9, P < 0.001; time x shoot removal F_23,48_ = 4.7, P = 0.01) (Supplementary Table 1).

**Table 1:** Mean viable and total length (±standard errors) of ERM produced by *Funneliformis mosseae* IMA1 and *Rhizoglomus irregulare* IMA6 in symbiosis with *Cichorium intybus* plants, at different time points after host shoot removal (detached roots) or in intact control plants (intact roots).

Time (days)	Viable ERM length (m)		Total ERM length (m)	
IMA1	Detached roots	Intact roots	Detached roots	Intact roots
0	6.4 ± 0.7 d*	6.4 ± 0.7 d	7.1 ± 1.7 d*	7.1 ± 1.7 c
30	12.9 ± 0.3 cd	13.2 ± 1.2 cd	16.3 ± 0.5 c	16.4 ± 1.2 c
60	20.3 ± 2.4 bc	20.9 ± 1.9 abc	26.3 ± 2.9 b	26.7 ± 2.8 b
90	23.4 ± 1.1 b	23.4 ± 1.6 ab	31.0 ± 1.3 b	31.3 ± 1.5 b
120	20.6 ± 2.1 bc	19.5 ± 2.4 bc	32.4 ± 2.0 b	34.0 ± 3.0 b
150	34.5 ± 2.6 a	28.1 ± 1.9 a	56.6 ± 0.8 a	44.8 ± 1.4 a
One-way (time)	F_5,12_ = 29.0 P < 0.001	F_5,12_ = 19.9 P < 0.001	F_5,12_ = 108.5 P < 0.001	F_5,12_ = 46.1 P < 0.001
**IMA6**	**Detached roots**	**Intact roots**	**Detached roots**	**Intact roots**
0	8.8 ± 0.6	8.8 ± 0.6	10.3 ± 0.7	10.3 ± 0.7
30	24.8 ± 6.1	18.1 ± 2.6	31.0 ± 7.4	23.0 ± 3.3
60	24.4 ± 1.6	26.8 ± 3.8	31.6 ± 1.5	32.2 ± 6.0
90	17.7 ± 1.1	31.5 ± 2.5	25.6 ± 1.5	43.3 ± 3.8
120	28.2 ± 2.9	26.3 ± 3.3	43.7 ± 3.4	43.6 ± 3.1
150	22.8 ± 1.8	20.6 ± 0.2	40.8 ± 2.8	33.1 ± 2.0
One-way (time)	F_5,12_ = 5.4 P = 0.008	Welch F_5,5_ = 4.9 P < 0.001	Welch F_5,5_ = 5.2 P < 0.001	F_5,12_ = 12.8 P < 0.001

*In columns, means followed by the same letter do not differ significantly at P ≤ 0.01 by Tukey’s HSD test.

Interestingly, in Exp. 2, the growth of *F. mosseae* IMA1 (hereafter IMA1) extraradical hyphae was continuous for the whole duration of the experiment (150 d) in both intact and shootless plants, and showed significant differences among the different time points (Table 1). Compared to ERM length at the beginning of Exp. 2, total length increases of 99 and 41% (90 days), 247 and 32% (150 days) were detected in ERM produced by IMA1 and *R. irregulare* IMA6 (hereafter IMA6), respectively. The extent of viable ERM in IMA1 mirrored the trend showed by total length, while in IMA6 it did not change with time (Table 1).

### Lifespan and structural traits of ERM spreading from roots of shootless plants

Microscopic observation of membranes obtained from Exp. 2, containing roots of intact or shootless plants, showed that after shoot removal and root death (occurring 4 days after shoot detachment), ERM maintained its structural traits. Histochemical localisation of SDH activity allowed the detection of protoplasmic continuity and viable anastomoses in hyphae growing from roots of both intact and shootless plants, ranging from 32 to 44% of total contacts, with no differences in interconnection rates among shootless/intact plants and fungal symbionts. ERM hyphae connected to dead roots maintained their viability and showed SDH activity for 5 months after shoot removal (Fig. 2d,e).

**Figure 3:**
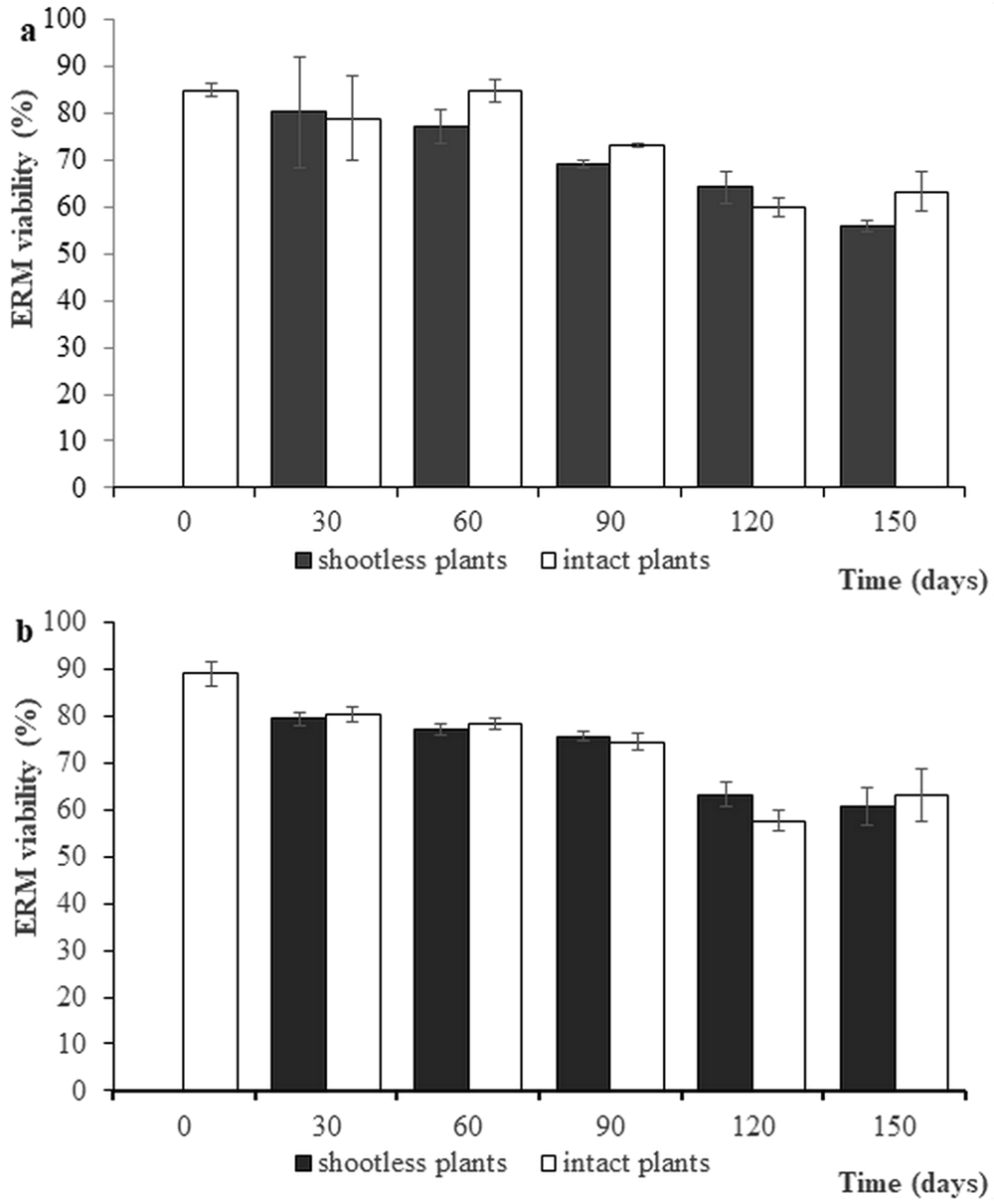
Percentage of viable extraradical mycelial length (means ± standard error of means) recorded in extraradical networks formed by *Funneliformis mosseae* IMA1 (**a**) and *Rhizoglomus irregulare* IMA6 (**b**) in symbiosis with *Cichorium intybus*, at variable times after shoot removal (shootless plants) or in intact plants.

In Exp. 2, the ratio between lengths of viable and total ERM connected to viable or dead roots showed constant decreases over time, with no significant differences among treatments and fungal symbionts (Fig. 3). Three-ways ANOVA with fungal species*shoot removal treatment*time for viable to total ERM length ratio showed that only time represented a significant factor of hyphal viability, without interactions among factors (F_11,24_ = 46.3, P < 0.001) (Supplementary Table 1). Regression analyses confirmed the reduction of ERM viability over time, with similar R^2^ values for mycelium spreading from roots of shootless (R^2^ = 0.81 and 0.78, for IMA1 and IMA6, respectively; P < 0.001) and intact plants (R^2^ = 0.75 and 0.53, for IMA1 and IMA6, respectively; P = 0.001) (Supplementary Figure S1a,b).

**Figure 4:**
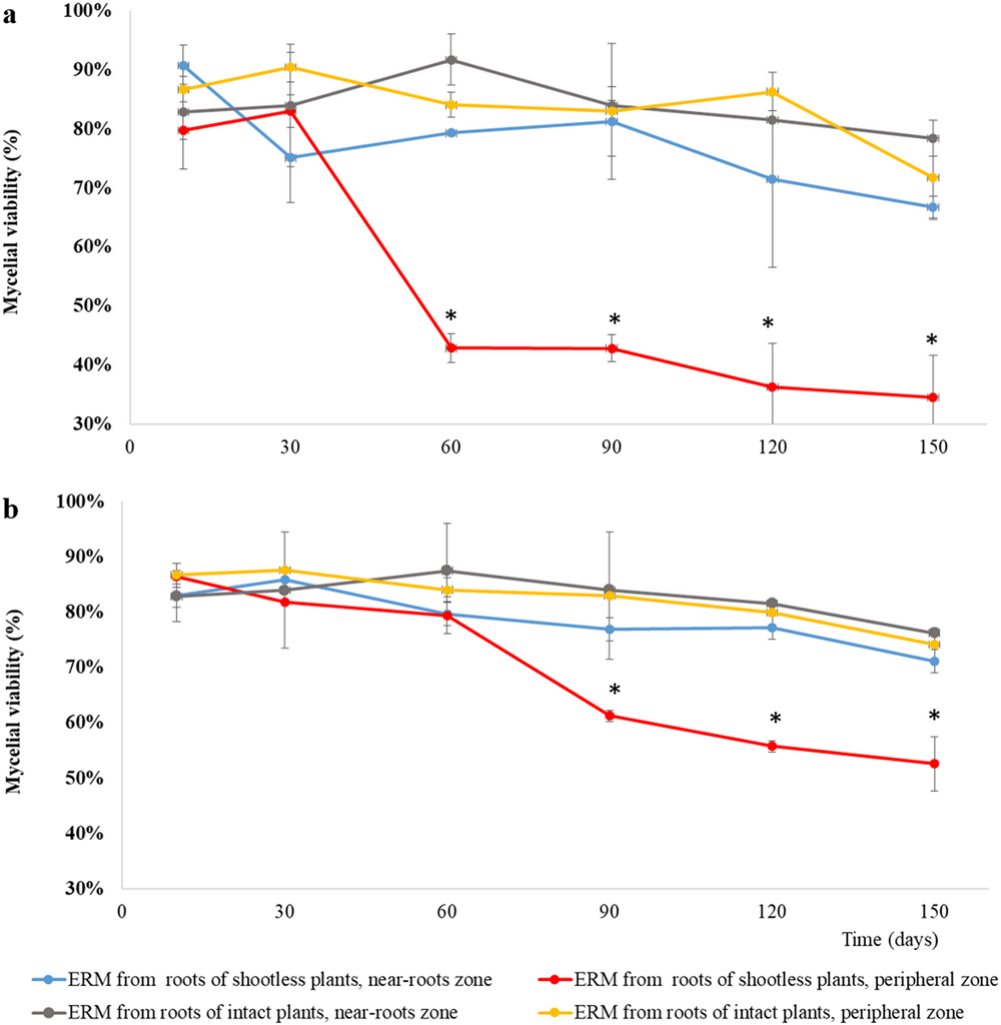
Percentage of viable extraradical mycelial length (means ± standard error of means) recorded close to roots (near-roots zone) or in the peripheral zone of extraradical networks formed by *Funneliformis mosseae* IMA1 (**a**) and *Rhizoglomus irregulare* IMA6 (**b**) in symbiosis with *Cichorium intybus*, at variable times after shoot removal (shootless plants) or in intact plants. At each time point, asterisks indicate viability values of ERM produced by roots of shootless plants which differ significantly (P < 0.01) from those of ERM produced by roots of intact plants.

**Figure 5:**
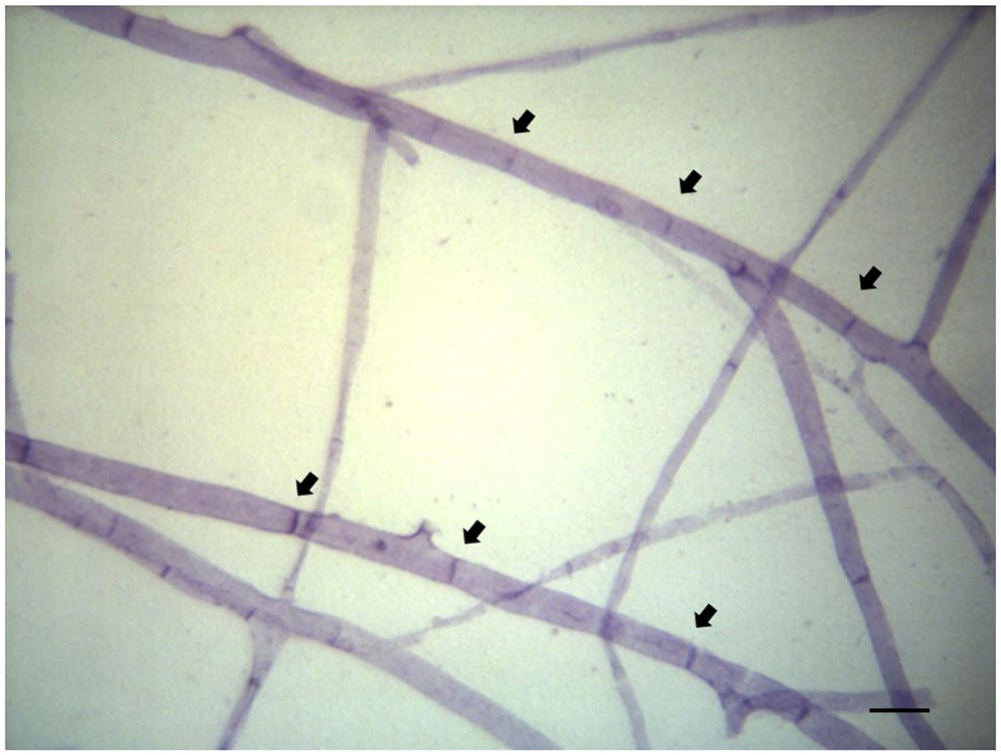
Light micrograph showing retraction septa (arrows) formed in peripheral hyphae devoid of protoplasm (succinate dehydrogenase activity staining) of extraradical mycelium spreading from *Funneliformis mosseae* IMA1 colonised roots, 4 months after *Cichorium intybus* shoot detachment; scale bar = 10 µm.

In Exp. 2, the viability of IMA1 mycelium connected to roots of intact plants did not differ among regions proximal to roots (1 cm far from roots) or distant from them (1 cm from the ERM edge), while a dramatic viability reduction over time was found in ERM regions distant from roots of shootless plants (Fig. 4a). In these samples, large ERM peripheral areas showed hyphal protoplasm withdrawal, empty segments and frequent retraction septa (crosswalls) as early as 60 days after shoot removal (Figs. 4a, 5). Considering time, shoot removal treatment and distance from roots as 3-ways ANOVA factors, all factors and their interactions exerted significant effects on IMA1 viable to total length ratio of ERM connected to roots of shootless plants (Supplementary Table 1), with significantly lower viability rates of peripheral zones of ERM connected to such roots compared with that originating from those of intact plants (Fig. 4a). Regression analyses showed higher correlation between viability and time in peripheral zones of ERM connected to dead roots (R^2^ = 0.72, P < 0.001) compared with the near-root zone (R^2^ = 0.36, P = 0.005), whereas the correlation was low or not significant for data obtained from ERM produced by intact plants (Supplementary Figure S1c).

In Exp. 2, data on the viability of IMA6 mycelium proximal or distant from roots were consistent with those obtained in IMA1, with a similar viability decrease in peripheral hyphae connected to dead roots (Fig. 4b). ERM proximity to roots, time since shoot removal, shoot removal treatment and their interactions (with the exception of 3-ways interaction) were significant in the analysis of viability of ERM (Supplementary Table 1). These data were confirmed by regression analyses of viability of ERM in peripheral areas over time (R^2^ = 0.92, P < 0.001) (Supplementary Figure S1c).

In both AMF isolates, viability recorded in peripheral areas of shootless plants ERM at 10 and 30 d time points (ranging from 79.7 to 86.5%) were significantly different from those detected at 90, 120 and 150 d time points (ranging from 34.5 to 61.2%) (Fig. 4).

### Colonisation ability of ERM spreading from roots after shoot removal

Results obtained from Experiment 3 showed that ERM growing from roots of shootless plants maintained its ability to establish mycorrhizal symbioses, as all chicory seedlings placed in contact with developed ERM were colonised at all time points (Table 2). Interestingly, chicory seedlings showed 40 and 70% root colonisation when in contact with ERM produced by IMA1 and IMA6, respectively, 4 months after host shoot removal. At the same time, colonised root length of chicory seedlings in contact with ERM produced by IMA1 and IMA6 in symbiosis with intact plants reached 57.5 ± 2.2% and 38.3 ± 13.9%, respectively. Mycorrhizal colonisation of seedling roots in contact with ERM connected to dead roots showed significant decrease or not over time, depending of AMF identity (IMA1: F_3,8_ = 14.11, P < 0.001, IMA6 not significant) (Table 2).

**Table 2:** Percentage of colonised seedlings (confidence limits 66.96–100%) and mycorrrhizal root length of *Cichorium intybus* plants maintained for 10 days in contact with *Funneliformis mosseae* IMA1 and *Rhizoglomus irregulare* IMA6 ERM spreading from detached roots at variable time points after plant shoot removal (n.a. indicate not available data).

	Percentage of colonised plants		Mycorrhizal root length (%)	
	IMA1	IMA6	IMA1	IMA6
10	100	100	55.8 ± 4.0 bc*	52.6 ± 8.2 a
60	100	100	92.9 ± 2.4 a	58.5 ± 5.8 a
90	100	100	88.5 ± 5.1 ab	69.1 ± 20.4 a
120	100	100	40.2 ± 15.0 c	70.2 ± 20.6 a
150	100	n.a.	28.8 ± 5.0 c	n.a.

*In columns, means (±standard error of mean) followed by the same letter do not differ significantly at P ≤ 0.001 by Tukey’s HSD test.

## Discussion

The data obtained in this work revealed that the cellular viability and functionality of extraradical hyphae are uncoupled from the host plant lifespan in two Glomeracean AMF isolates. For the first time, we showed that, after shoot removal, the growth of ERM spreading from detached root systems was comparable with that from intact plants, and continuous for the whole duration of the experiment, 150 d. Moreover, ERM showed comparable and high levels of viability, and maintained its ability to establish mycorrhizal symbioses with new plants, as long as 5 months after shoot removal. These findings expand the current basic knowledge on AMF biology and life cycle and on ERM role in the maintenance of mycorrhizal potential in the soil.

Shoot removal did not interfere with ERM growth, which was continuous and uninterrupted up to 5 months, mirroring ERM length from roots of intact plants. This interesting result may be explained by the release of nutrients from dead roots and subsequent uptake by AMF hyphae. Indeed, root cell membranes may increase their permeability during senescence and boost the transfer of nutrients from the host to the fungus^29^, in agreement with data reporting that in *Lolium perenne* roots detached from the shoots lost up to 60% of their initial N and up to 70% of their P within the first three weeks^30^. Previous works investigated the impact of plant shoot removal on ERM nutrient transfer activity in plant interconnected by AMF extraradical hyphae and showed significant transfer of N and P from dying donor roots to receiver plants after shoot removal from the donor plant^31,32^. Accordingly, Müller *et al*.^33^ found significant translocation of ^15^N from dead donor roots to receiver plants in mycorrhizal tomato. Such findings represent evidence of the persistence of ERM viability and functionality in dead root systems, recorded over short periods of time, 2 and 6 weeks, respectively. As our work lasted 5 months, we could suppose that AMF hyphae were able to recapture from detached roots not only nutrients early released after shoot removal, and not only N and P, but also all the other nutrients they need for the continuous growth detected here. Indeed, arbuscules, the main sites where nutrient exchanges between the two partners of AM symbiosis occur, were viable in dead roots 5 months after shoot removal, suggesting that they might be still able to uptake nutrients from host roots and to support ERM life, thus leading to the maintenance of soil mycorrhizal inoculum potential after crop harvest (Fig. 2)^24,34^.

Here, the viability of extraradical hyphae spreading from roots of shootless plants was monitored for a time longer than in previous experiments, and showed metabolic activity for 5 months after shoot removal. In experiments using a comparable system, other authors found 100% viability of the mycorrhizal networks after 7 days^3^, while the metabolic activity of extraradical mycelium in the soil showed values of 63% and 100% in 6-weeks-old and 3-weeks-old *Glomus intraradices* and *Glomus clarum* hyphae, respectively^35,36^. Other works reported that the length of viable extraradical mycelium was 20–40 m m^−1^ colonised root in *Eucalyptus coccifera*, depending on AMF identity^37^. It is interesting to note that ERM viability was higher in hyphae proximal to roots, while a dramatic viability reduction was found in hyphae distant from roots of shootless plants. Such findings are consistent with previous observations on the occurrence of degraded-looking cytoplasm or empty and septated zones in old ERM developed by *Gigaspora rosea* and *Glomus intraradices in vitro*^38^ and with the described behaviour of AMF germling hyphae, whose metabolic activity declined steeply with increasing distance from the spore, where distal and older hyphal tips appeared empty, dead and showed frequent retraction septa^39^.

Our data, for the first time, showed that when the shoots were detached from the roots, the extraradical hyphae not only continued to grow at the same rate as the ones spreading from intact plants, but they also maintained the same structural traits, *i.e*. a high interconnectedness by means of frequent anastomoses. This is an important finding, as the preservation of the structure allowing a continuous flow of nutrients within the hyphal network may further explain the observed growth increase of hyphae spreading from detached roots.

*F. mosseae* IMA1 and *R. irregulare* IMA6 hyphae spreading from dead roots were able to colonise new host plants and establish mycorrhizal symbioses, even 5 months after shoot removal. Other authors have previously reported that hyphae of different AMF species were able to survive and grow from dead root fragments to colonise new host roots, suggesting that such hyphae may play an important role as propagules for rapid host colonisation after soil disturbance^28,40^, also following prolonged freezing^41,42^. Contrary to AMF species belonging to Glomerales, those belonging to the family Gigasporaceae were not found able to colonise new plants (*Gigaspora* spp.) or gave inconsistent results (*Scutellospora calospora*) when inoculum was represented by dead roots, suggesting a differential behaviour among AMF, possibly related with their sensitivity to disturbance^28,43,44^.

Hyphal ability to survive, grow and retain host infection capability independently from the host plant lifespan may represent an important trait, functional to the conservation and increase of mycorrhizal potential of soils. Indeed, ERM produced by native AMF was able to survive Mediterranean summer dry conditions, maintaining its colonisation ability^34^, while soil mycorrhizal potential and diversity was enhanced by mycotrophic cover crops^24,45,46^, as the result of the development of extensive mycelial networks, retaining their viability and infectivity after harvest. On the other hand we observed a similar decrease in average viability in ERM connected to both intact and shootless plants at 150 days of growth: it would be interesting to investigate whether ERM reduced viability over time, even when connected to intact plants, due to changes in plant nutrient transfer ability occurring in aged hosts.

In conclusion, our findings offer a new vision of AMF life cycle in the soil, where fungal extraradical mycelium, growing from both living and dead roots, represents a long-term survival structure - beyond quiescent spores and mycorrhizal root fragments - functional to the prompt establishment of mycorrhizal symbioses and to the maintenance of soil biological fertility and mycorrhizal inoculum potential in agricultural soils.

## Materials and Methods

### Experimental design

An *in vivo* experimental design was devised in order to obtain intact AMF extraradical hyphae developing from mycorrhizal roots, to be monitored after the detachment of host plant shoots^47^. Time-course experiments allowed the study of lifespan and structural traits of ERM connected to roots of shootless plants, as well as its colonisation ability (mycorrhizal potential). A schematic outline of the experimental design is presented in Supplementary Figure S2. *Cichorium intybus* seeds were surface-sterilised, germinated and grown for 10 days in sterile quartz grit and then inoculated with 50 mg (fresh weight) of sieved inoculum. Such inoculum, composed of spores or sporocarps, mycelium and colonised roots, was obtained from pot-culture soil of two different AMF, *Funneliformis mosseae* isolate IMA1 and *Rhizoglomus irregulare* isolate IMA6 (in the text, IMA1 and IMA6), which was suspended in water and sieved (repeating this procedure up to 10 times) through a 100-µm-mesh size sieve. Inoculated plants were grown in 10-cm diameter pots filled with sterile quartz grit, in a growth chamber with 25 °C day and 21 °C night temperature, 16 h of light per days. After four weeks’ growth, grit was washed from roots, which were checked for the absence of contaminating fungal mycelium or propagules other than AMF, and spores and sporocarps adhering to plant roots, along with most extraradical hyphae, were carefully removed with forceps under a Leica M 205 C dissecting microscope (Leica, Milano, Italy) and root systems were singly wrapped in nylon nets (40 µm mesh)^47^. Each root system was then placed between two semicircular 13-cm diameter Millipore™ membranes (root sandwiches), transferred into 14-cm diameter Petri dishes containing moist sterile quartz grit, with the root-containing lower half of plates wrapped into aluminium foil, and maintained in the growth chamber^47^. After ten days, plants which showed 12–15 cm^2^ of the membrane areas covered by ERM (assessed with a transparent graph paper) were selected for the different experiments, and the shoots of plants of the “shootless plant” treatment were cut with sterile blades (1 cm over the upper edge of membrane sandwiches), without disturbing the root systems and the mycorrhizal extraradical networks growing on the membranes, while plants belonging to the “intact plant” treatment were not affected (Supplementary Figure S2). Plates with membrane sandwiches containing root systems of both intact and shootless plants were placed back in the growth chamber.

### Experiment 1. Viability of plant roots after shoot removal

To evaluate root viability maintenance after shoot detachment, 12 plants of *C. intybus* were germinated, inoculated with *F. mosseae* isolate IMA1, grown, placed in the membrane system and then shoots were removed as described above. Uninoculated shootless controls were prepared using the same procedure and timing, but no inoculum was added to the seedlings. After shoot removal, the viability of uninoculated and inoculated roots was checked daily on three replicates of inoculated and control root systems for 4 d using 10 µg ml^−1^ fluorescein diacetate (FDA) (Sigma-Aldrich s.r.l. Milan, Italy) in phosphate buffer (0.1 M, pH 7.4)^48^. This staining is a cell-permeant esterase substrate that can serve as a viability probe indicating both enzymatic activity, which is required to activate the fluorescence, and cell-membrane integrity, which is required for intracellular retention of the fluorescent product^48^. The roots were stained for 10 min at 21 °C, rinsed in the same buffer and observed under the Reichert-Jung Polyvar epifluorescent microscope equipped with excitation filter BP 450–495, barrier filter LP 520 and dichroic mirror DS 510. The proportion of viable and total root length was assessed on three aliquots (each of 4 × 3 cm root length) for each root system by the gridline intersect method^49^, using a grid eyepiece at 100× magnification.

### Experiment 2. Lifespan and structural traits of ERM spreading from roots after shoot removal

A total of 36 replicates (18 shootless plants and 18 control intact plants) for each AMF isolate were prepared and sequentially harvested 0, 30, 60, 90, 120 and 150 days after shoot removal (three replicates per time point, per AMF and per treatment). At each time point, membranes of sandwich systems containing *F. mosseae* IMA1 and *R. irregulare* IMA6 ERM connected to roots of shootless and intact plants were stained for the localisation of succinate dehydrogenase (SDH) activity^50^ followed by Trypan blue in lactic acid (0.05%). On each membrane, check of roots and intraradical fungal structures viability and assessment of viable and total ERM hyphal density were carried out under the dissecting microscope. ERM hyphal densities (hyphal length mm^−2^) were assessed on three replicate membranes at each time point and for each treatment, by the gridline intersect method, using a microscope eyepiece grid at 320 × magnification^47^. On each replicate membrane two different measures of both viable and total hyphal density were compared to examine ERM viability: (i) in six randomly selected areas of 9 mm^2^, (ii) in six areas of 9 mm^2^ within 1 cm from nylon-enclosed roots and in six areas selected within 1 cm from the edge of ERM. Moreover, the area explored by ERM was assessed by using a transparent 5-mm-square grid. Total and viable hyphal lengths were calculated from measured data, multiplying mean hyphal densities by the area explored by the mycelial network. Areas of membranes (10 × 25 mm each) covered by ERM were cut, mounted on microscope slides and examined under a Reichert-Jung (Vienna, Austria) Polyvar microscope. ERM hyphal contacts were counted at x320 magnification on at least three samples from each replicate membrane and perfect fusions were recorded when hyphae anastomosed and protoplasm continuity could be verified by SDH staining.

### Experiment 3. Colonisation ability of ERM spreading from roots after shoot removal

To monitor the maintenance of colonisation ability by ERM, 15 replicate shootless plants of mycorrhizal *C. intybus* for each AMF isolate were obtained using the experimental system described above. Three sandwiches for each AMF were carefully opened at different time points (10, 60, 90, 120 and 150 days after shoot removal) and two *C. intybus* seedlings were inserted in the sandwich, placing their roots on ERM spreading from the roots (Supplementary Figure S2). After 10 days of incubation, colonised length of the inserted seedling roots was assessed after Trypan blue staining, using the gridline intersect method at 40× magnification^47^. To compare the colonisation ability of ERM from shootless root systems with that of ERM from intact plants, three replicates of control sandwich systems for each AMF, containing intact mycorrhizal plants of *C. intybus*, were obtained as described, and two new *C. intybus* seedlings were inserted in each control sandwich in parallel with the 120 days time point of shootless plants.

### Data analysis

Data were checked for fulfilment of ANOVA assumptions (by Shapiro-Wilk and Levene’s tests) and three and two-ways ANOVA were performed to assess the significance of the different factors involved (fungal identity, intact/shootless plant treatment, time and ERM distance from roots) on fungal parameters (viability rates and length of ERM) and on plant root colonisation. Percentage data were analysed after arcsin transformation. One-way ANOVA followed by Tukey’s HSD multiple range test (homogeneous variances) or Welch’s test (not-homogeneous variances) was used to detect significant differences among data obtained for each AMF isolate and plant treatment (different time points) or for each isolate and time point (different plant treatments). Correlation and regression analyses were carried out to reveal relationships among fungal parameters and time and between ERM viability and colonisation ability. All statistical analyses were performed with SPSS Statistics version 23 (IBM Corp., Armon, NY Inc, USA).

### Data availability

The datasets analysed during the current study are available from the corresponding author on reasonable request.

## Electronic supplementary material

Supplementary file
