# Supplementary material for: Lifespan and functionality of mycorrhizal fungal mycelium are uncoupled from host plant lifespan
Source: Sci Rep. 2018 Jul 6;8:10235. doi: 10.1038/s41598-018-28354-5 (PMC6035242; doi:10.1038/s41598-018-28354-5)
Supplement: Supplementary file 1 — Supplementary file [file 41598_2018_28354_MOESM1_ESM.pdf]

Lifespan and functionality of mycorrhizal fungal mycelium are uncoupled from host plant lifespan

Alessandra Pepe, Manuela Giovannetti, Cristiana Sbrana

Supplementary Table S1 a, b. Summary of three-way ANOVA testing the effects of AMF identity, shoot removal treatment and time since shoot removal on ERM total and viable lengths and on viable to total length ratio (a) and the effects of ERM distance from roots, shoot removal treatment and time since shoot removal on ERM viable to total length ratio of each fungal isolate (a).

**a**	**Source of variation**	**df**	**Mean total ERM length**		**Mean viable ERM length**		**Mean viable/total ERM ratio**	
			F	P	F	P	F	P
	AMF	1	**7.8**	**0.008**	**6.6**	**0.014**	0.5	0.47
	shoot removal	1	0.2	0.62	0.0	0.91	0.5	0.48
	time	5	**78.3**	**0.000**	**36.1**	**0.000**	**46.3**	**0.000**
	AMF*shoot removal	1	0.7	0.39	1.1	0.29	1.0	0.32
	AMF*time	5	**10.0**	**0.000**	**7.8**	**0.000**	1.3	0.28
	shoot removal*time	5	**4.7**	**0.001**	**3.0**	**0.02**	1.3	0.29
	AMF*shoot removal*time	5	2.3	0.06	2.2	0.07	0.4	0.81

**b Viable/total ERM ratio**	**Source of variation**	**df**	***Funneliformis mosseae* IMA1**		***Rhizoglomus irregulare* IMA6**	
			F	P	F	P
	shoot removal	1	**131.6**	**0.000**	**50.4**	**0.000**
	ERM distance from roots	1	**50.9**	**0.000**	**13.3**	**0.001**
	time	5	**19.2**	**0.000**	**21.4**	**0.000**
	shoot removal* ERM distance from roots	1	**51.7**	**0.000**	**11.9**	**0.001**
	shoot removal*time	5	**8.9**	**0.000**	**4.2**	**0.003**
	ERM distance from roots*time	5	**8.2**	**0.000**	**3.7**	**0.007**
	shoot removal* ERM distance from roots*time	5	**3.3**	**0.01**	2.4	0.052

Lifespan and functionality of mycorrhizal fungal mycelium are uncoupled from host plant lifespan

Alessandra Pepe, Manuela Giovannetti, Cristiana Sbrana

Supplementary Figures

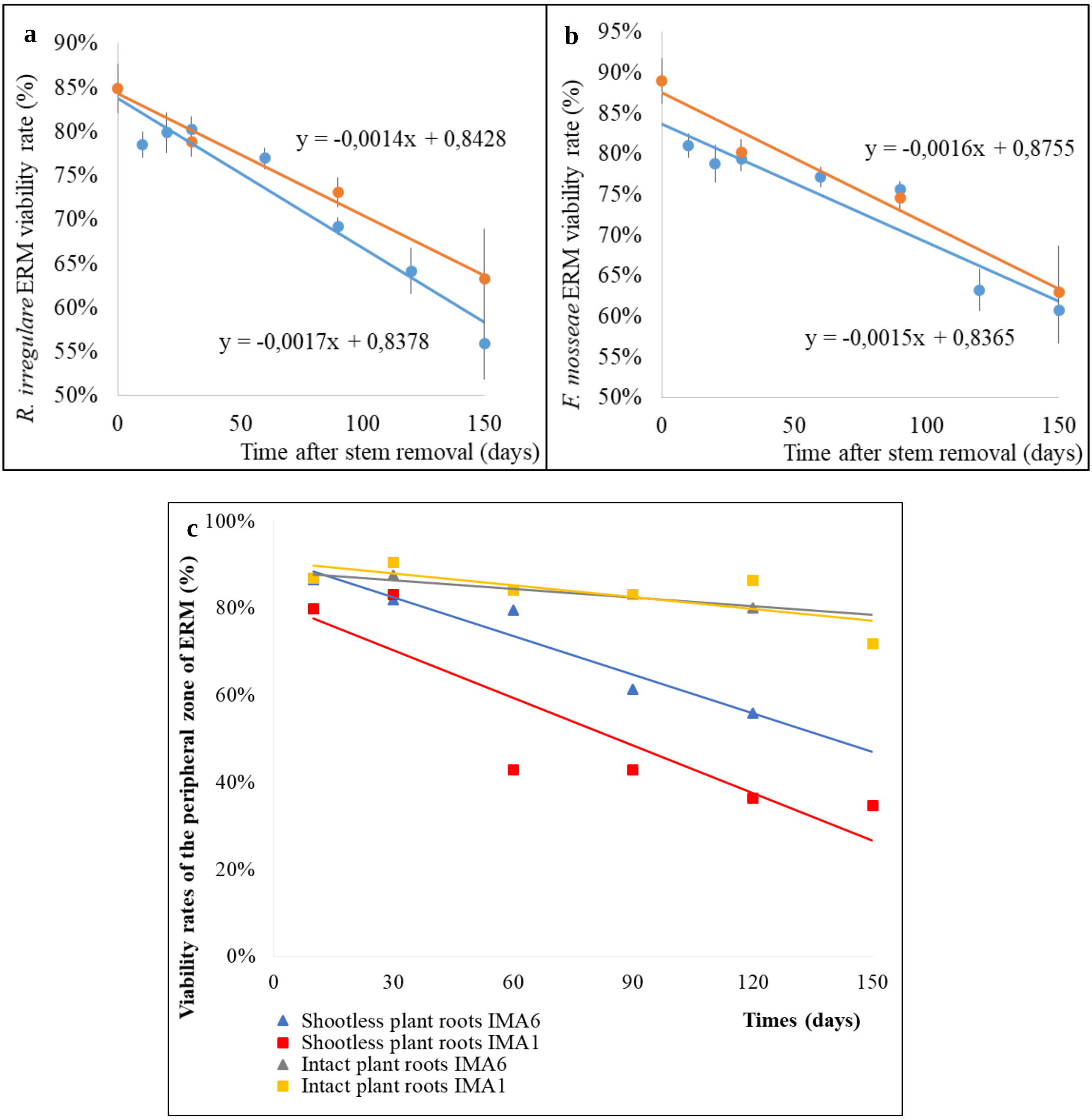

Figure S1. a, b: linear regression computed for data of mean viability rates recorded in ERM produced by *Funneliformis mosseae* IMA1 (a) and *Rhizoglomus irregulare* IMA6 (b) spreading from roots of intact (blue) and shootless (orange) *Cichorium intybus* plants; **c:** linear regression computed for viability rates data, recorded in peripheral areas of ERM produced by *F. mosseae* IMA1 and *R. irregulare* IMA6 spreading from roots of intact and shootless *C. intybus* plants.

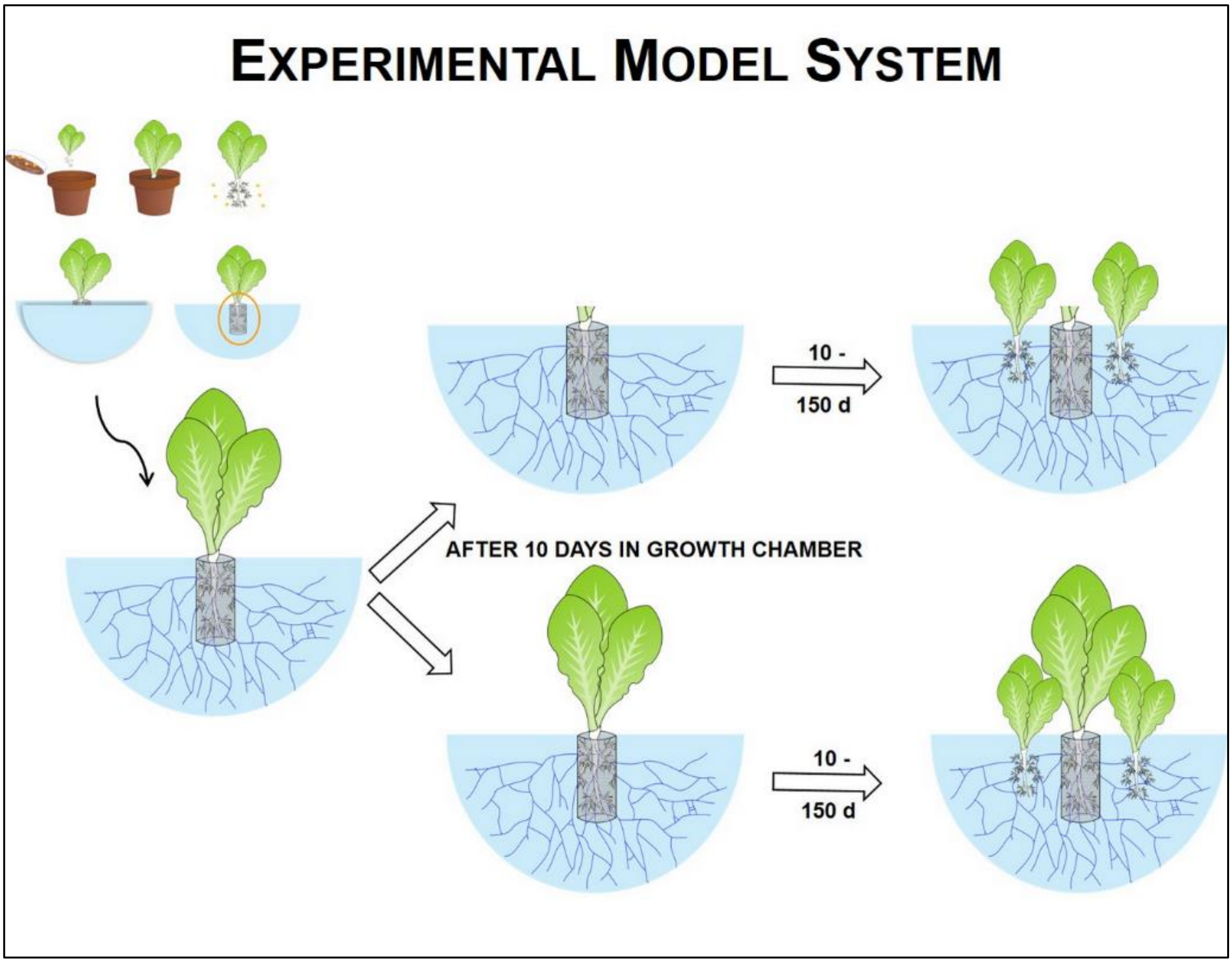

Figure S2. Schematic representation of the experimental system used to assess the viability and colonisation ability of extraradical mycelium connected to roots of intact or shootless plants. *Cichorium intybus* germlings grown in sterile quartz grit were inoculated with pot-culture soil sieving of *Funneliformis mosseae* isolate IMA1 and *Rhizoglomus irregulare* isolate IMA6 and maintained in pots filled with sterile quartz grit in growth chamber. After four weeks' growth, grit was washed from roots and extraradical hyphae and spores were removed with forceps, root systems wrapped in nylon nets and placed between two 13-cm diameter membranes (root sandwiches). Plants were then transferred in Petri dishes with sterile quartz grit on both sides of root sandwich and maintained in the growth chamber. After ten days, shoots were detached, without disturbing the root systems. At different time points after shoots detachment, new *Cichorium intybus* seedlings were inserted in the sandwiches, placing their roots on extraradical hyphae connected to living or dead root systems.
